# Large extracellular vesicles in the left atrial appendage in patients with atrial fibrillation—the missing link?

**DOI:** 10.1007/s00392-021-01873-4

**Published:** 2021-06-01

**Authors:** Andreas Zietzer, Baravan Al-Kassou, Paul Jamme, Verena Rolfes, Eva Steffen, Marko Bulic, Mohammed Rabiul Hosen, Philip Roger Goody, Vedat Tiyerili, Sebastian Zimmer, Jan Wilko Schrickel, Alexander Sedaghat, Bernardo S. Franklin, Nikos Werner, Georg Nickenig, Felix Jansen

**Affiliations:** 1 Medical Department II, University Hospital Bonn, Venusberg-Campus 1, 53127 Bonn, Germany; 2 Institute of Innate Immunity, Medical Faculty, University of Bonn, Venusberg-Campus 1, 53127 Bonn, Germany; 3 Medizinische Klinik III, Krankenhaus der Barmherzigen Brüder Trier, Nordallee 1, 54292 Trier, Germany; 4 Medizinische Klinik und Poliklinik II, Innere Medizin, Kardiologie, Pneumologie und Angiologie, Universitätsklinikum Bonn, Venusberg-Campus 1, 53127 Bonn, Germany

**Keywords:** Extracellular vesicle, Microvesicle, Platelet, Atrial fibrillation, microRNA-222-3p, microRNA-223-3p, Endothelial function

## Abstract

Atrial fibrillation (AF) is the most frequent arrhythmic disease in humans, which leads to thrombus formation in the left atrial appendage and stroke through peripheral embolization. Depending on their origin, large extracellular vesicles (lEVs) can exert pro-coagulant functions. In the present study, we investigated how different types of AF influence the levels of large EV subtypes in three distinct atrial localizations. Blood samples were collected from the right and left atrium and the left atrial appendage of 58 patients. 49% of the patients had permanent AF, 34% had non-permanent AF, and 17% had no history of AF. Flow cytometric analysis of the origin of the lEVs showed that the proportion of platelet-derived lEVs in the left atrial appendage was significantly higher in permanent AF patients compared to non-permanent AF. When we grouped patients according to their current heart rhythm, we also detected significantly higher levels of platelet-derived lEVs in the left atrial appendage (LAA) in patients with atrial fibrillation. In vitro studies revealed, that platelet activation with lipopolysaccharide (LPS) leads to higher levels of miR-222-3p and miR-223-3p in platelet-derived lEVs. Treatment with lEVs from LPS- or thrombin-activated platelets reduces the migration of endothelial cells in vitro. These results suggest that permanent atrial fibrillation is associated with increased levels of platelet-derived lEVs in the LAA, which are potentially involved in LAA thrombus formation.

**Supplementary Information:**

The online version contains supplementary material available at 10.1007/s00392-021-01873-4.

## Introduction

Atrial fibrillation (AF) is the most frequent arrhythmic cardiac disease in humans and a significant health burden across the world [1, 2]. AF is characterized by unorganized ectopic electrical activity in the atrium, which is caused by an atrial tissue substrate (typically local damage) [3]. While genetic susceptibility plays a certain role in the development of AF [4, 5], it is primarily the remodeling of the atrial tissue that is both the cause and consequence of further arrhythmic episodes [6]. This inflicts a vicious circle of disease progression which starts with few, usually self-limiting, arrhythmic episodes (paroxysmal AF) but almost inevitably leads to permanent AF, if no treatment is administered [7]. The most serious consequence of AF is thrombus formation in the left atrium, which typically takes place in the left atrial appendage (LAA). Along with stasis of the blood in the LAA, impaired endothelial function has been shown to be an important trigger and a hallmark for atrial thrombus formation in atrial fibrillation [8–10]. Embolization of these thrombi into the cerebral arteries leads to stroke and makes AF the most important cause of embolic stroke [11, 12]. In this context, several studies have pointed out, that the risk of stroke in AF patients depends on the AF burden over time. This means that patients with permanent AF have a higher risk of stroke than patients with non-permanent AF [13, 14]. Recently, this concept has been challenged, because it is still not completely understood which factors are involved in atrial thrombus formation in AF [15].

In the context of thrombus formation, extracellular vesicles (EVs) and in particular platelet-derived EVs have been shown to be potent inductors of coagulation [16, 17]. EVs are membranous bodies that are released by various cell types and serve as a means of intercellular communication [18, 19]. One of the most significant determinants of the biological function of EVs is the levels of encapsulated microRNAs. These microRNAs can be transferred to and taken up by other cells and influence their biological behavior [20]. EVs are traditionally grouped into large EVs (formerly called microvesicles) and small EVs (formerly called exosomes). As large EVs can be analyzed directly by flow cytometry, we have focused on large EVs (lEV) for this study. A number of studies have found differences in the levels of EVs from different origins in the peripheral blood between non-AF and AF patients [21–24]. It has therefore been assumed that EVs are involved in the formation of atrial thrombi in AF patients. However, a specific analysis showing differences in thrombogenic EV levels in the left atrial appendage across different types of AF has not been performed.

In this study, we hypothesized that the subtype of AF (non-permanent vs. permanent) influences the level of pro-coagulatory platelet-derived lEVs in the LAA, which could serve as a path to investigate why thrombus formation and stroke are more common in permanent AF than in non-permanent AF patients.

## Material and methods

### Patient recruitment

For this analysis, 58 patients undergoing invasive procedures requiring venous access and transseptal puncture at the University Hospital Bonn were included. The recruitment took place from February 2018 until July 2019. Each patient underwent right and left atrial catherization for individual medical indications after written and informed consent had been obtained. Besides withdrawal of consent no further exclusion criteria were applied. The majority of the patients underwent mitral valve reconstruction, while few patients were treated with an occluder system of the LAA (Table 1). In all cases the catheter system was introduced via the right femoral vein. Directly after the transseptal puncture, unfractionated heparin was administered at a dose of 70–100 IE/kg bodyweight to reach an activated clotting time of 250–300 s. For the definition of different types of AF, we used a simplified version of the 2016 ESC Guidelines [25]. In the first group, we included patients, who have never had AF. In the second group, we included patients with permanent AF without any reported attempt to restore a sinus rhythm for at least one year. In the third group, we included patients with intermittent episodes of AF but, who did not fulfill the criteria for permanent AF. In order to define coronary artery disease, the definition of the American heart association from 2013 was used: (at least one 50% stenosis in the diameter of a major coronary artery) [26]. All the MitraClip patients underwent coronary angiography in preparation for the intervention. For the six remaining patients, the medical history was screened for coronary angiography reports. Echocardiographic routine measurements were performed in the facilities of the heart center at the University Hospital of Bonn. Aortic valve stenosis, mitral valve insufficiency and mitral valve stenosis were classified following recommendations of the European society of cardiology [27–29]. All echocardiographic data were reviewed by two independent cardiologists. The study was approved by the ethics committee of the University Hospital of Bonn (283/16) and is in accordance with the Declaration of Helsinki.

**Table 1:** Baseline characteristics in different types of AF

		All patients (*n* = 58)	No AF (*n* = 10)	Non-perm. AF (*n* = 21)	Perm. AF (*n* = 27)	*p* value Chi-squared test/ANOVA + Tukey’s test
Age (years)		78.8 ± 7.1	77.4 ± 6.4	78.9 ± 6.0	79.2 ± 8.3	0.8023
Male sex (*n*, %)		31 (53)	6 (60)	10 (47.6)	15 (55.6)	0.7759
BMI (kg/m^2^)		26.2 ± 4.9	24 ± 3.9	25.7 ± 5.0	27.4 ± 4.9	0.1446
Heart rate at the procedure (bpm)		67.9 ± 14.1	67.0 ± 11.3	64.1 ± 12.9	71.4 ± 15.6	0.2411
**Current intervention**						
Implantation of MitraClip (n, %)		52 (89.7)	9 (90)	18 (85.7)	25 (92.6)	0.7393
Implantation of left atrial appendage occluder system (n, %)		5 (8.6)	0 (0)	3 (14.3)	2 (7.4)	0.3967
Mitral balloon valvuloplasty (n, %)		1 (1.7)	1 (10)	0 (0)	0 (0)	0.0870
**Cardiovascular risk factors**						
Hypertension (*n*, %)		56 (96.6)	10 (100)	20 (95.2)	26 (96.3)	0.7901
Diabetes mellitus (n, %)		14 (24.1)	5 (50)	1 (4.8)	8 (29.6)	**0.0150**
		**No vs. non-perm: *p* = 0.0029; Non-perm vs. perm: *p* = 0.0285**				
Dyslipidemia (*n*, %)		52 (89.7)	8 (80)	20 (95.2)	24 (88.9)	0.4215
Familial burden (*n*, %)		6 (10.3)	0 (0)	4 (19)	2 (7.4)	0.2101
Smoking (*n*, %)		12 (20.7)	2 (20)	3 (14.3)	7 (25.9)	0.6130
**Medical history**						
Coronary artery disease (n, %)		32 (55.2)	7 (70)	10 (47.6)	15 (55.6)	0.5028
Previous myocardial infarction (n, %)		15 (25.9)	3 (30.0)	5 (23.8)	7 (25.9)	0.9345
Previous coronary bypass (n, %)		10 (17.2)	3 (30.0)	3 (14.3)	4 (14.8)	0.5014
Previous TAVR (n, %)		6 (10.3)	0 (0.0)	1 (4.8)	5 (18.5)	0.1492
Peripheral artery disease (n, %)		15 (25.9)	5 (50.0)	2 (9.5)	8 (29.6)	**0.0459**
		**No vs. non-perm: *p* = 0.0117**				
Congestive heart failure NYHA III + IV (*n*, %)		45 (77.9)	7 (70.0)	18 (85.7)	20 (74.5)	0.5168
**Echocardiography**						
Left ventricular ejection fraction (%)		51.73 ± 1.98	49,65 ± 5.82	50,91 ± 3.05	53,38 ± 2.85	0.7601
Mitral valve regurgitation grade III + IV (*n*, %)		41 (74.6)	8 (80.0)	16 (76.2)	17 (70.9)	0.4716
Mitral valve stenosis > grade I (*n*, %)		5 (8.6)	1 (10.0)	1 (4.8)	3 (11.5)	0.6882
Aortic valve stenosis > grade I (*n*, %)		0 (0.0)	0 (0.0)	0 (0.0)	0 (0.0)	
Left atrial size (mL)		92.6 ± 7.3	70.0 ± 4.5	88.0 ± 8.9	107.1 ± 14.9	0.2025
**Key medications at baseline **						
Antiplatelet drugs (n, %)		23 (39.7)	7 (70)	7 (33.3)	9 (33.3)	0.0978
Vitamin K antagonists (VKA) (n, %)		15 (25.9)	2 (20)	4 (19)	9 (33.3)	0.4786
Non-VKA oral anticoagulants (n, %)		32 (55.2)	2 (20)	16 (76.2)	14 (51.9)	**0.0118**
		**No vs. non-perm: *p* = 0.0030**				

Data are displayed as *n* (%) or mean ± SD

For the in vitro experiments, platelets were isolated from peripheral venous blood of healthy volunteers after written and informed consent had been obtained. The study was approved by the ethics committee of the University Hospital of Bonn (282/17) in accordance with the Declaration of Helsinki.

### Preparation of blood and isolation of EVs

**Fig. 1:**
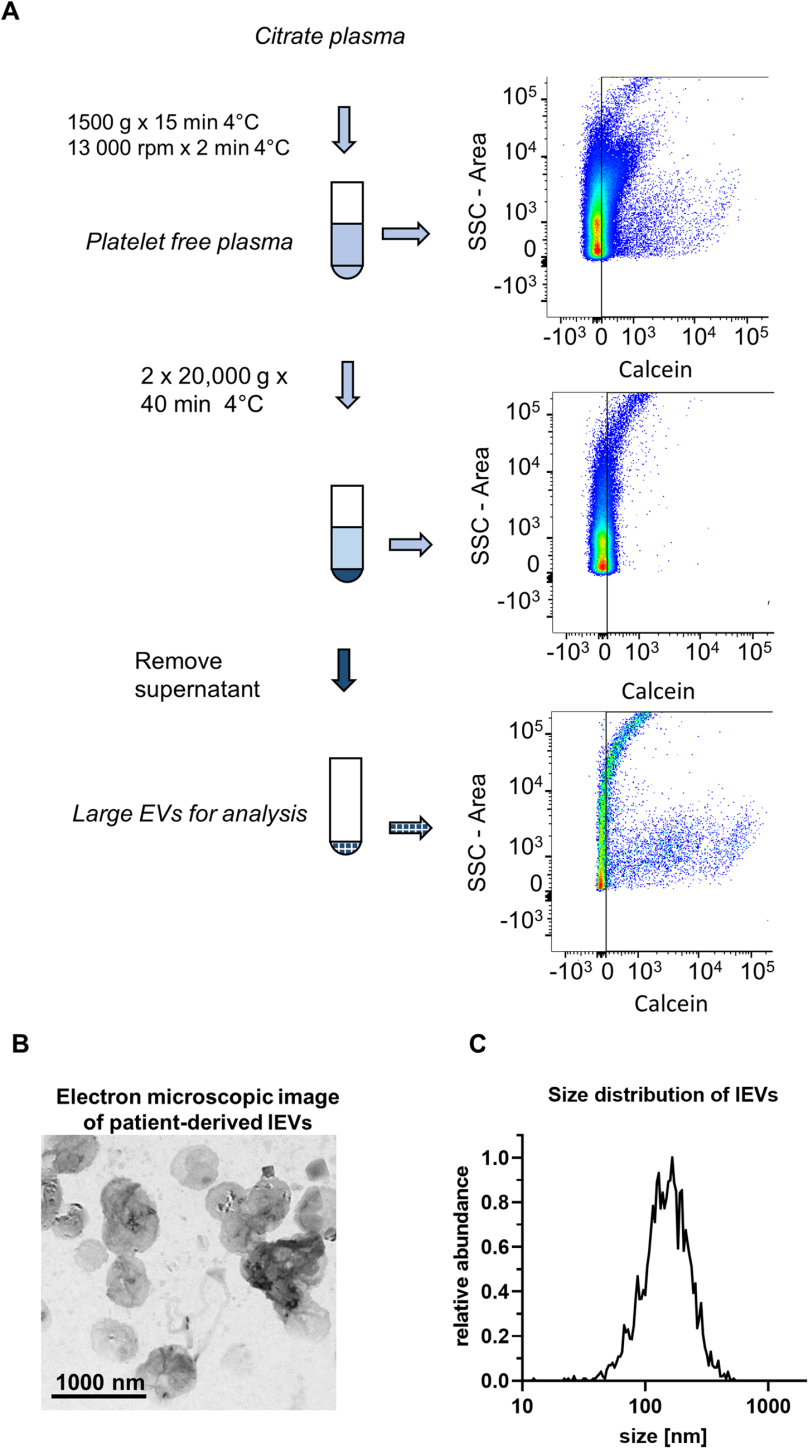
**a** Diagram of lEV isolation procedure with representative FACS analysis at different steps of the isolation. **b** Electron microscopic imaging lEVs by use of negative staining. **c** lEV size distribution as measured by nanoparticle tracking analysis

For the clinical analyses, all patients had fasted for at least 6 h before the catheter intervention. Blood was drawn through the catheter system from the right atrium (RA), the left atrium (LA), and the LAA. The blood was collected in trisodium citrate buffer to give a final citrate concentration of 3.2%. Cells were immediately removed from the blood using a two-step centrifugation protocol with 1,500 × *g* for 15 min and 13,000 rpm for 2 min, as previously reported [30, 31]. The platelet-free plasma was then stored at − 80 °C until further analyses were performed. EVs were isolated by centrifugation at 20,000 × *g* for 40 min with an additional washing step with PBS (Fig. 1). Further routine analyses, such as measurements of total cholesterol, HDL cholesterol, LDL cholesterol, triglycerides, lipoprotein a, creatinine, C-reactive protein, white blood cells, red blood cells, hemoglobin, and platelets were performed from additional venous blood samples in the central laboratory facilities of the Institute of Clinical Chemistry and Clinical Pharmacology at the University Hospital Bonn. Routine coagulation activity measurements including INR, aPTT, Factor II, Factor V, Factor VII, Factor VIII, and Factor X were performed at the facilities of the Institute for Experimental Hematology und Transfusion Medicine (IHT) at the University Hospital Bonn.

For the in vitro experiments, platelets were isolated from peripheral venous blood as previously reported [32]. In brief, other blood cells were removed by a two-step centrifugation at 330 × *g* for 5 min and at 340 × *g* for 10 min. Prostaglandin E1 (PGE1; 200 nM) was used to inhibit platelet activation in the second step and for all subsequent centrifugation steps. Finally, the platelets were pelleted at 430 × *g* for 15 min. After counting in a hemocytometer, the platelets were resuspended in RPMI medium to achieve a concentration of 5 × 10^7^ cells per ml.

### Flow cytometric analysis of lEV

For flow cytometric analysis, the lEVs from 150 µl of platelet-free plasma were used. The isolated lEVs were resuspended in 100 µl PBS and then incubated with 1 µl anti-CD31-PE (BD Pharmingen, Cat# 555446), 1 µl anti-CD41-APC (BD Pharmingen, Cat# 559777), and 1 µl anti-CD235-PE-Vio770 (Miltenyi Biotec, Cat# 130-120-614) antibodies for 45 min at RT. For specific staining of intact lEVs, 0.5 µL of 1 mM Calcein AM (Thermo Fisher Scientific, Cat# C1430) in DMSO was added together with 400 µl sterile PBS [33]. Following a 20 min incubation at RT, 50 µl of AccuCount Blank Particles 2.0–2.4 µm (Spherotech, Cat# ACBP-20-10) were added for counting. A FACSCanto II (BD Bioscience) was used for sample analysis. Compensation, gating, and absolute quantification were performed with the software FlowJo V10 (BD Bioscience). To determine if the Calcein-stained particles are intact membranous bodies, 0.5 µL Triton X-100 (Sigma-Aldrich, Cat# T8787) were added to the sample after the last staining step to degrade the lEVs. This resulted in a Triton-X100 concentration close to 1% in the final sample. A swarming effect (detection of multiple EVs as one event) was excluded by use of a dilution series ranging from 50 to 250 µl platelet-free plasma as the input for the analysis [34]. To assess the size of the plasma-derived EVs, Silica Microspheres, 1.0 μm (Polysciences Cat# 24326–15) and Silica Microspheres, 0.3 μm (Polysciences Cat# 24321–15) were used, as previously reported [35]. In brief, 0.5 μl of the bead solutions were added to 200 μl PBS and analyzed directly.

### Nanoparticle tracking analysis

The size distribution of plasma-derived lEVs was assessed by nanoparticle tracking analysis in a ZetaView BASIC NTA—Nanoparticle Tracking Video Microscope PMX-120 (Particle Metrix). To this end, EVs were isolated from 500 µL plasma and finally resuspended in 500 μL PBS. The samples were then further diluted 1:250 in PBS and analyzed. The concentration was confirmed to be in the linear range of the instrument.

### Electron microscopy of lEV

Electron microscopic imaging was performed as previously reported [36]. In brief, lEVs isolated from 1 ml plasma were finally resuspended in 10 µL PBS with Protease Inhibitor Cocktail (Roche, Cat# 4693132001). Prior to loading, the lEVs were diluted further 1:250 in PBS and 5 µl of the suspension were loaded on Formvar-coated copper grids (Science Services, München). After incubation for 20 min at RT, the sample was then fixed for 5 min with 2% paraformaldehyde. Subsequently, the EVs were washed with PBS and fixed again for 5 min with 1% glutaraldehyde. Finally, the sample was contrast stained with 1.5% uranyl acetate for 4 min. Image acquisition was performed with a Jem-2100Plus (Jeol) operating at 200 kV using a Gatan OneView 4 K camera.

### Platelet stimulation and lEV isolation

The isolated platelets were stimulated with 200 ng/ml LPS or 1.0 U/ml thrombin or left untreated (native) for 3 h. Subsequently, the platelets were removed from the suspension by centrifugation at 3000 × *g* for 10 min. The effective removal of platelets was confirmed by microscopic visualization in a hemocytometer. The supernatants were immediately frozen at −80 °C until further use. For the isolation of in vitro-generated large platelet-derived EVs (abbreviated pEV, to distinguish them from patient-derived platelet EVs), the supernatants were thawed on ice and pEVs were pelleted by centrifugation at 20,000 × *g* for 40 min, washed with PBS, and pelleted again.

### RNA isolation

For the isolation of RNA from pEVs, pEVs isolated from 500 μL of platelet supernatant were resuspended in 250 μL RNAse-free water before 750 μL Trizol LS were added (Invitrogen, Cat# 10296010). We used *Caenorhabditis elegans* miR‐39 (cel‐miR‐39) RNA at a final concentration of 5 nmol/L (Qiagen) as a spike-in control, as previously reported [36]. To isolate RNA from the HCAEC samples, the cells were directly dissolved in Trizol (Invitrogen, Cat# 15596026) after washing with ice-cold PBS. RNA extraction with chloroform, isopropanol, and ethanol was conducted as previously reported [31]. The RNA was dissolved in pure H_2_O. Purity (260/280 ratio > 1.8) and concentration of the isolated RNA were assessed with a NanoDrop2000 spectrophotometer (Thermo Fisher Scientific) as previously reported [37, 38].

### microRNA quantification with TaqMan assays by qPCR

10 ng of total RNA were used for cDNA preparation with the TaqMan microRNA Reverse Transcription kit (Applied Biosystems, Cat# 4366596), adhering to the manufacturer’s protocols. 1 µL of the resulting cDNA solution was used for quantitative real-time PCR with the respective TaqMan probe (hsa-miR-126-3p Cat# 4427975, Assay ID 002228; hsa-miR-222-3p, Cat# 4427975, Assay ID 000525; hsa-miR-223-3p, Cat# 4427975, Assay ID 000526; cel-miR-39-3p, Cat# 4427975, Assay ID 000200; RNU6b, Cat# 4427975, Assay ID 001093) and TaqMan Universal Master Mix II (Applied Biosystems, Cat# 4440040) in a 7500 HT Real-Time PCR instrument (Applied Biosystems). MiR levels in pEVs were calculated as 2^−ddCT^ vs. the spike-in control cel-miR-39. For the experiments with HCAECs, cellular miR-expression was calculated as 2^−ddCT^ vs RNU6b as an internal control.

### Scratch-wound assay

To assess the effect of pEVs on EV recipient cells, we used endothelial cells, because endothelial cells are the only resident cells in the LAA that are in direct contact with blood-derived EVs. As endocardial endothelial cells from the LAA are not commercially available, we used female primary human coronary artery endothelial cells (HCAECs) from passage 8 (Promocell, Cat# C-1222) for basic migration experiments in a scratch-wound assay. The assay was performed as previously described [39]. In brief, HCAECs were allowed to grow to confluency in a 6-well plate. The pEVs from 500 µl of supernatant were resuspended in 500 µl fresh RPMI medium and further diluted 1:1 in Endothelial Cell Growth Medium MV with supplements (Promocell, Cat# C-22020) before incubation with the HCAECs. Directly after stimulation, a scratch was applied to the center of the well with a sterile 200 µl pipet tip. The scratch was photographed in a marked position at 0, 2, 4, and 6 h. To calculate the migratory activity of the cells, the remaining cell-free area was measured and compared to the cell-free area at 0 h. Image acquisition and analyses were conducted with a Zeiss Axio Observer microscope and the ZEN 2.3 pro software.

### Transfection of miRs into HCAECs

To simulate an overexpression of miR-222-3p and miR-223-3p in HCAECs, we used miR-222-3p-mimic, miR-223-3p-mimic, or control RNA (all Invitrogen: miR-222-3p mirVana miRNA mimic, Cat# 4464066, Assay ID MC11376; mir-223-mirVana miRNA mimic, Cat# 4464084, Assay ID MC24077, mirVana miRNA mimic Negative Control 1, Cat# 4464060). The RNAs were transfected at a final concentration of 10 nmol/L by use of Lipofectamine RNAiMAX Transfection reagent (Invitrogen, Cat# 13778150) within 24 h of incubation. Effective overexpression of the miRs was confirmed via qPCR. The Wound-scratch assays were started 24 h after the transfection.

### Statistical analysis

Statistical analyses were performed using the software Prism8 (GraphPad). The lEV data in Fig. 3 were analyzed by a two-way ANOVA followed by Tukeys’s multiple comparison test. The distribution of the data in Fig. 4 differed significantly from a normal distribution as assessed by the D’Agostino and Pearson’s test. Therefore, a Mann–Whitney *U* test was used to analyze the data in Fig. 4. For the in vitro experiments, means of two groups were compared with an unpaired t-test and means of more than two groups were compared by a one-way ANOVA followed by Tukeys’s multiple comparison test. Categorial variables in the baseline characteristics were compared with the Chi-squared test. The number of independent experiments as well as the applied tests for statistical significance are reported in the figure legends. All reported *p* values are two-sided. The data presented in this study are available from the corresponding author upon request.

## Results

For the flow cytometric analysis of blood-derived lEVs, we included 58 patients with a mean age of 78.8 ± 7.1 years, equally distributed between both sexes (47% female 53% male) and with a mean body mass index of 26.2 ± 4.9 kg/m^2^ (Table 1). The patients were divided into three groups: (i) patients without any history of AF, (ii) patients with non-permanent AF, and (iii) patients with permanent AF [25]. The heart rate of the patients did not differ significantly at the time of the sample collection between the groups of AF. No differences between these groups were detected for cardiovascular risk factors, with the exclusion of diabetes mellitus, which showed a significantly higher prevalence in the no AF group compared to the other two groups. When considering the patients’ medical history, the prevalence of peripheral artery disease was significantly higher in the no AF group compared to the permanent AF group. No differences were detected for the prevalence of coronary artery disease, previous myocardial infarction, coronary bypass, transfemoral aortic valve replacement, congestive heart failure (NYHA III + IV). Routine echocardiographic imaging revealed a tendency towards larger LA volumes in patients with permanent AF (Table 1). The differences were not statistically different. No differences were found for mitral valve regurgitation (grade III + IV), mitral valve stenosis (grade > I) or left ventricular ejection fraction. No cases of aortic valve stenosis (grade > I) were detected in our collective. Also, when considering the drugs the patients had been taking that have a direct effect on coagulation and platelet activation, patients in the non-permanent AF group more frequently received non-VKA anticoagulants, while there was no difference for anti-platelet drugs. Routine laboratory parameters at baseline showed no differences for parameters of cardiovascular risk, inflammation, or kidney function (Table 2). However, the coagulation factors II, V, VII, and X were significantly decreased in the groups with AF, which was expected due to their higher rate of treatment with anticoagulants.

**Table 2:** Key laboratory parameters at time of inclusion in the study

	All patients (*n* = 58)	No AF (*n* = 10)	Non-perm. AF (*n* = 21)	Perm. AF (*n* = 27)	*p* value ANOVA + Tukey’s test
Total cholesterol [mg/dl]	154.9 ± 45.8	158.6 ± 49.3	158.5 ± 39.3	150.8 ± 50.4	0.8286
HDL-chol. [mg/dl]	50 ± 13.7	50.6 ± 14.3	53.1 ± 11.2	47.3 ± 15.1	0.3427
LDL-chol. [mg/dl]	94 ± 38.7	94.5 ± 36.8	88.9 ± 32.1	97.8 ± 44.5	0.7392
Triglycerides [mg/dl]	99.0 ± 52.8	96.7 ± 61.8	110.8 ± 55.3	90.9 ± 47.8	0.4653
Lipoprotein a [nmol/l]	61.6 ± 104.8	89.2 ± 66.9	79.3 ± 159.4	35.4 ± 50.9	0.3635
Creatinine [mg/dl]	1.55 ± 1.04	1.87 ± 1.14	1.35 ± 0.58	1.59 ± 1.26	0.4201
C-reactive protein [mg/l]	15.91 ± 24.31	21.46 ± 31.09	16.77 ± 27.3	13.07 ± 19.20	0.6512
INR []	1.3 ± 0.4	1.2 ± 0.3	1.2 ± 0.3	1.5 ± 0.5	0.0544
aPTT [s]	29.2 ± 5.3	27.4 ± 3.4	29.1 ± 6.3	30 ± 4.9	0.4276
White blood cells [10^9^/l]	7.1 ± 2.3	6.7 ± 2.3	7.7 ± 2.7	6.9 ± 1.8	0.3866
Red blood cells [10^12^/l]	3.7 ± 0.7	3.8 ± 0.8	3.7 ± 0.7	3.7 ± 0.5	0.7942
Hemoglobin [g/dl]	11.1 ± 2.2	11 ± 2.3	11.3 ± 2.4	11.0 ± 2.1	0.8917
Platelets [10^9^/l]	208 ± 64.2	200 ± 65.2	218 ± 72.3	202.4 ± 57.2	0.6658
Factor II [%]	75.1 ± 25.8	84.1 ± 17.8	83.2 ± 26.4	63.8 ± 24.7	**0.0183**
	**Non-perm. vs. perm. *p* = 0.0289**				
Factor V [%]	93.5 ± 24.4	109.3 ± 33.4	96.2 ± 22.8	84.1 ± 17	**0.0166**
	**No vs. perm. *p* = 0.0150**				
Factor VII [%]	71.2 ± 31	84.4 ± 33.6	81.4 ± 29	56.2 ± 26	**0.0067**
	**No vs. perm. *p* = 0.0322; Non-perm. vs. perm. *p* = 0.0145**				
Factor VIII [%]	202.2 ± 60.6	227.6 ± 62.6	203.8 ± 60.5	189.7 ± 58.7	0.2560
Factor X [%]	72 ± 31.2	78.9 ± 27.4	81.9 ± 29.8	59.9 ± 31.1	**0.0448**
	**Non-perm. vs. perm. *p* = 0.0472**				

Data are displayed as *n* (%) or mean ± SD

**Fig. 2:**
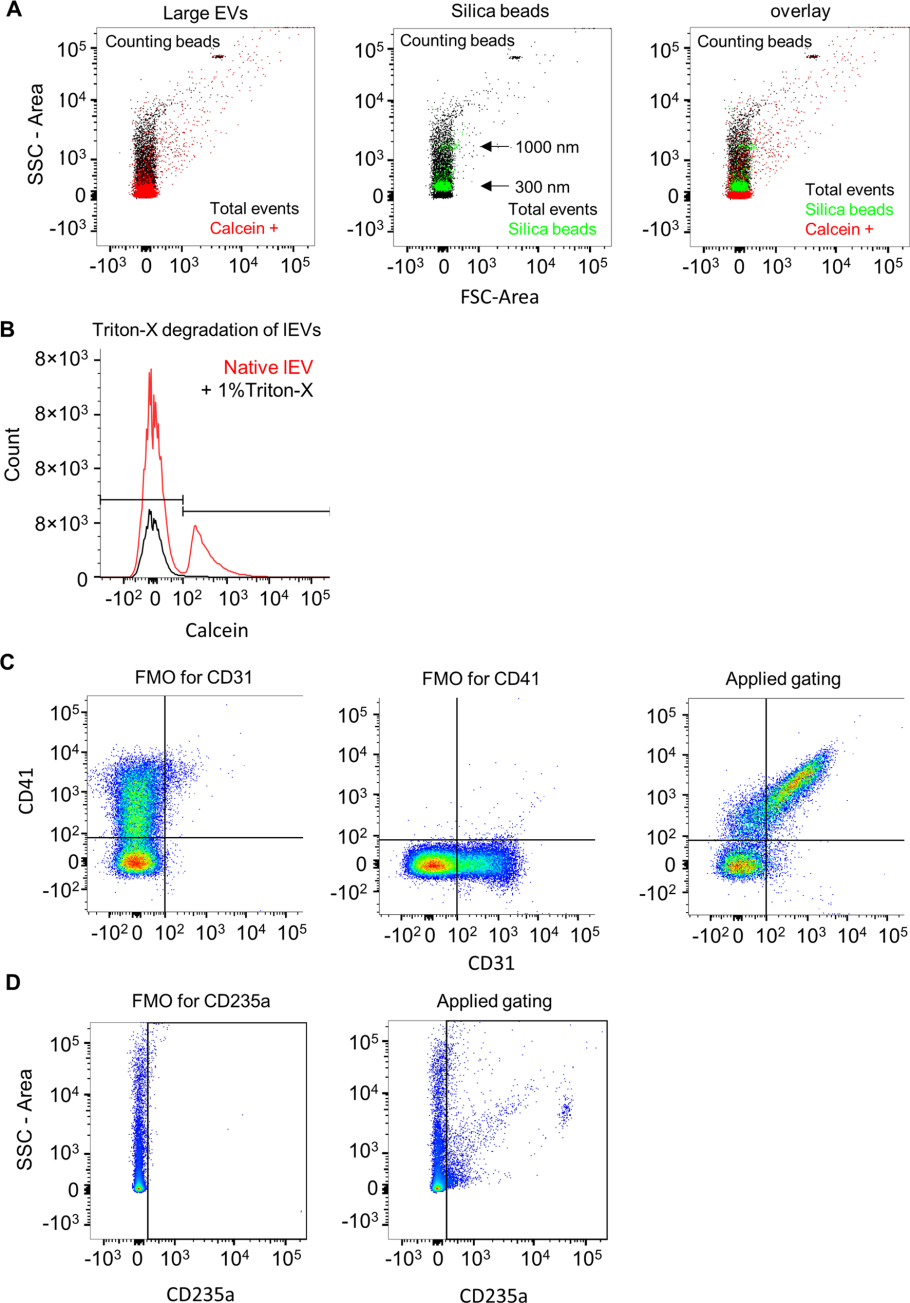
**a** Silica beads that have a 300 or 1000 nm diameter and a comparable refractory index as lEVs were used to estimate the size distribution of the lEVs by flow cytometry. **b** Effective staining of intact lEVs was confirmed by a lysis experiment with 1% Triton-X 100. **c**, **d** Gating strategy for the flow cytometric analyses of different lEV subtypes. Calcein AM-positive (Calcein^+^) events were identified as displayed in Fig. 1. Further gates were established by fluorescence-minus-one controls (FMO) for CD41-APC, CD31-PE, and CD235a-PE-Vio770. Platelet-derived EVs (Calcein^+^/CD41^+^/CD31^+^), endothelial cell-derived EVs (Calcein^+^/CD41^−^/CD31^+^), and red blood cell-derived EVs (Calcein^+^/CD235a^+^) were gated as illustrated

lEVs were isolated from citrate plasma from the three different atrial locations (described in the Methods section) of the patients and characterized by electron microscopy, nanoparticle tracking analysis, and flow cytometry. We found a size distribution ranging from 80 to 800 nm, with a peak around 200 nm (Figs. 1B, C, 2A). The specific staining of intact lEVs by Calcein AM was assessed in a lysis experiment, where 1% Triton-X was added just before analysis in the cytometer. After lysis with Triton-X, the lEV signal disappeared completely (Fig. 2B). To exclude a swarming effect, which is characterized by the detection of multiple lEVs as one event, we performed a dilution series by varying the amount of lEV input into the analysis. Increasing or decreasing the number of lEV input led to directly proportional changes in the detection of events, which suggests that swarming is not a factor (Figure S1). To further distinguish between different cellular EV subtypes, we applied the following gates: platelet-derived EVs (Calcein^+^/CD41^+^/CD31^+^), endothelial cell-derived EVs (Calcein^+^/CD41^−^/CD31^+^) (Fig. 2C), and red blood cell-derived EVs (Calcein^+^/CD235a^+^) (Fig. 2D).

**Fig. 3:**
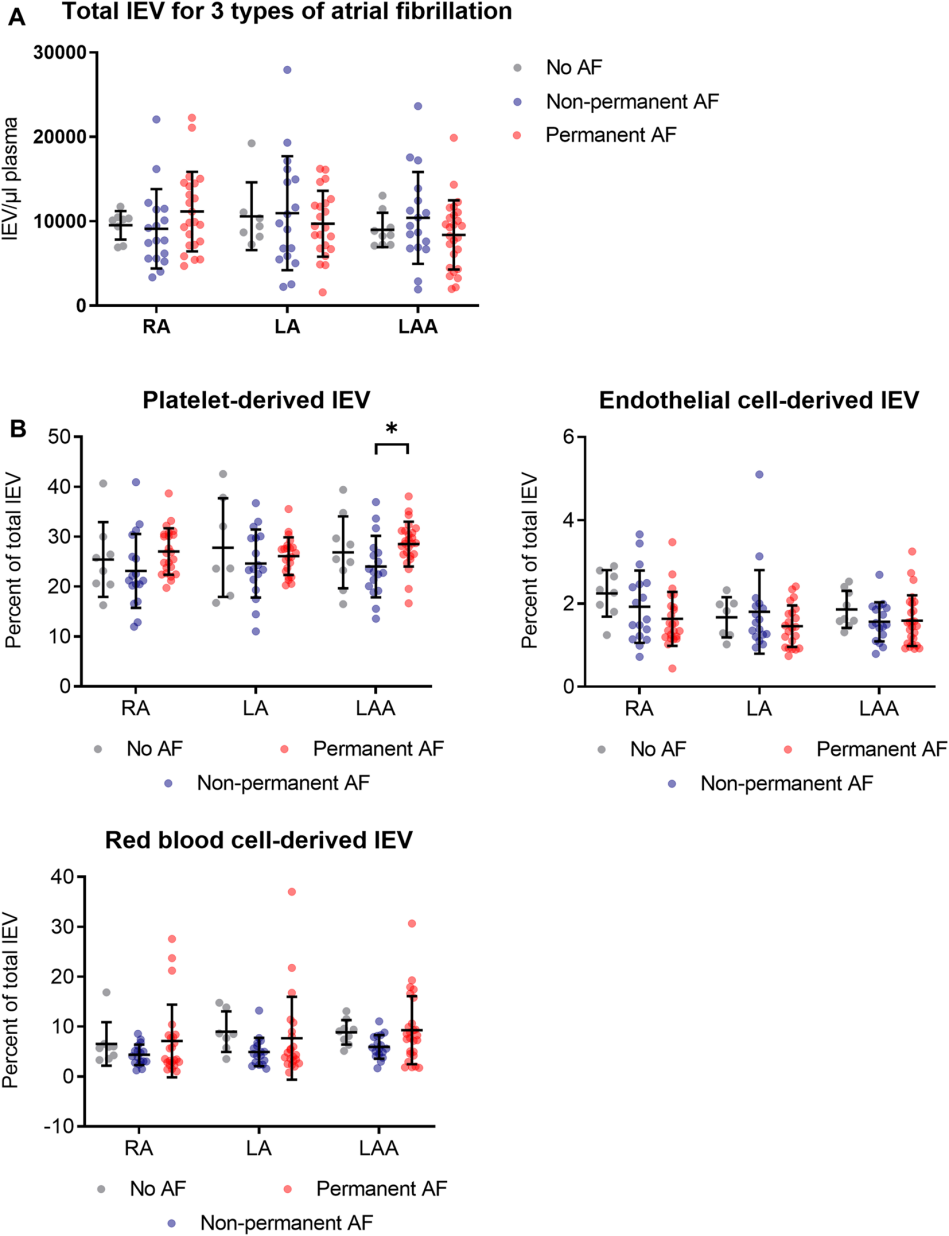
Flow cytometric analysis of lEVs in three atrial locations (*RA* right atrium, *LA* left atrium, *LAA* left atrial appendage) grouped by different types of atrial fibrillation (no history of AF, non-permanent AF, permanent AF). **a** Total lEV numbers per location (Calcein^+^ events) **b** Percentage of platelet-derived lEVs, endothelial cell-derived lEVs, and red blood cell-derived EVs at the three atrial locations in different types of atrial fibrillation. *n* = 10/21/27. Data are presented as individuals with the mean ± SD; *p < 0.05; two-way ANOVA + Tukey’s multiple comparison test

Quantification of the total number of EVs revealed no significant difference between the three groups of patients across the three atrial locations (Fig. 3A). With regard to the different EV subtypes, we found that exclusively in the LAA the relative portion of platelet-derived EVs was significantly higher in permanent AF patients compared to non-permanent AF. No changes were detected for endothelial cell-derived EVs or red blood cell-derived EVs across the three patient groups and locations (Fig. 3B).

**Fig. 4:**
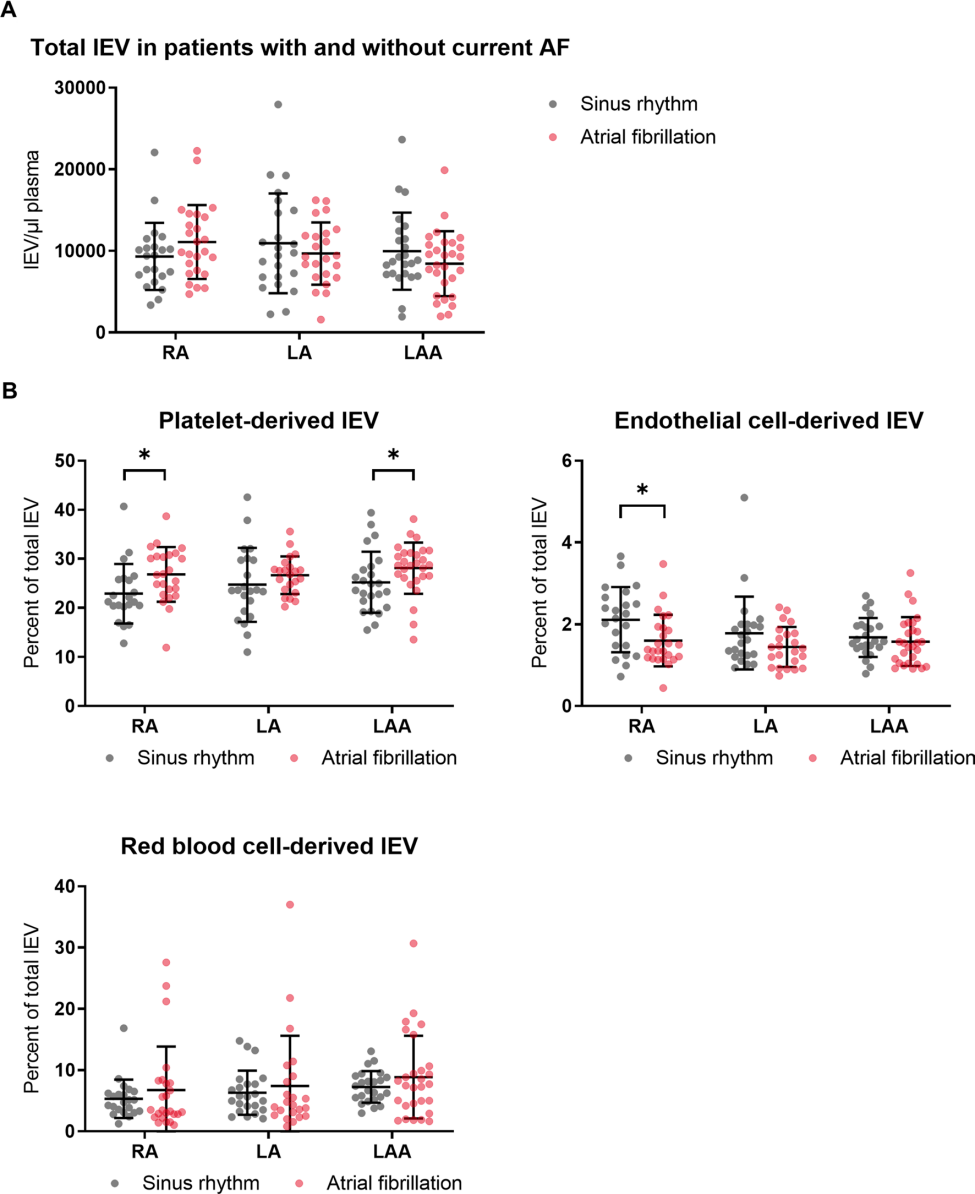
Flow cytometric analysis of lEVs in three atrial locations (RA, LA, LAA) grouped by current heart rhythm at the time of catheterization (sinus rhythm vs. atrial fibrillation). **a** Total lEV numbers per location (Calcein^+^ events) **b** Percentage of platelet-derived lEVs, endothelial cell-derived lEVs, and red blood cell-derived lEVs at the three atrial locations grouped by current heart rhythm. *n* = 10/21/27. Data are presented as individuals with the mean ± SD; *p < 0.05, Mann–Whitney *U* test

To confirm the finding that AF influences the distribution of lEV subtypes in the LAA, we regrouped our patients by current heart rhythm at the time of the blood collection (sinus rhythm vs. AF). Again, no significant differences were detected in total lEV numbers, but the relative portion of platelet-derived lEVs was significantly higher in the LAA and slightly lower in the RA in the AF group (Fig. 4A). Endothelial cell-derived lEVs were significantly lower in the RA of AF patients, whereas no difference was found for endothelial cell-derived lEVs in the LAA between the sinus-rhythm and AF groups (Fig. 4B). Red blood cell-derived lEVs were not significantly different between patients in sinus rhythm compared to AF patients for any of the atrial locations studied.

Interestingly, only platelet-derived lEV levels in the LAA were increased in AF patients (Fig. 3B), while total platelet levels were unchanged (Table 2). Therefore, we inferred that AF is associated with platelet activation, leading to increased levels of platelet-derived lEVs in the LAA, as previously reported [40].

**Fig. 5:**
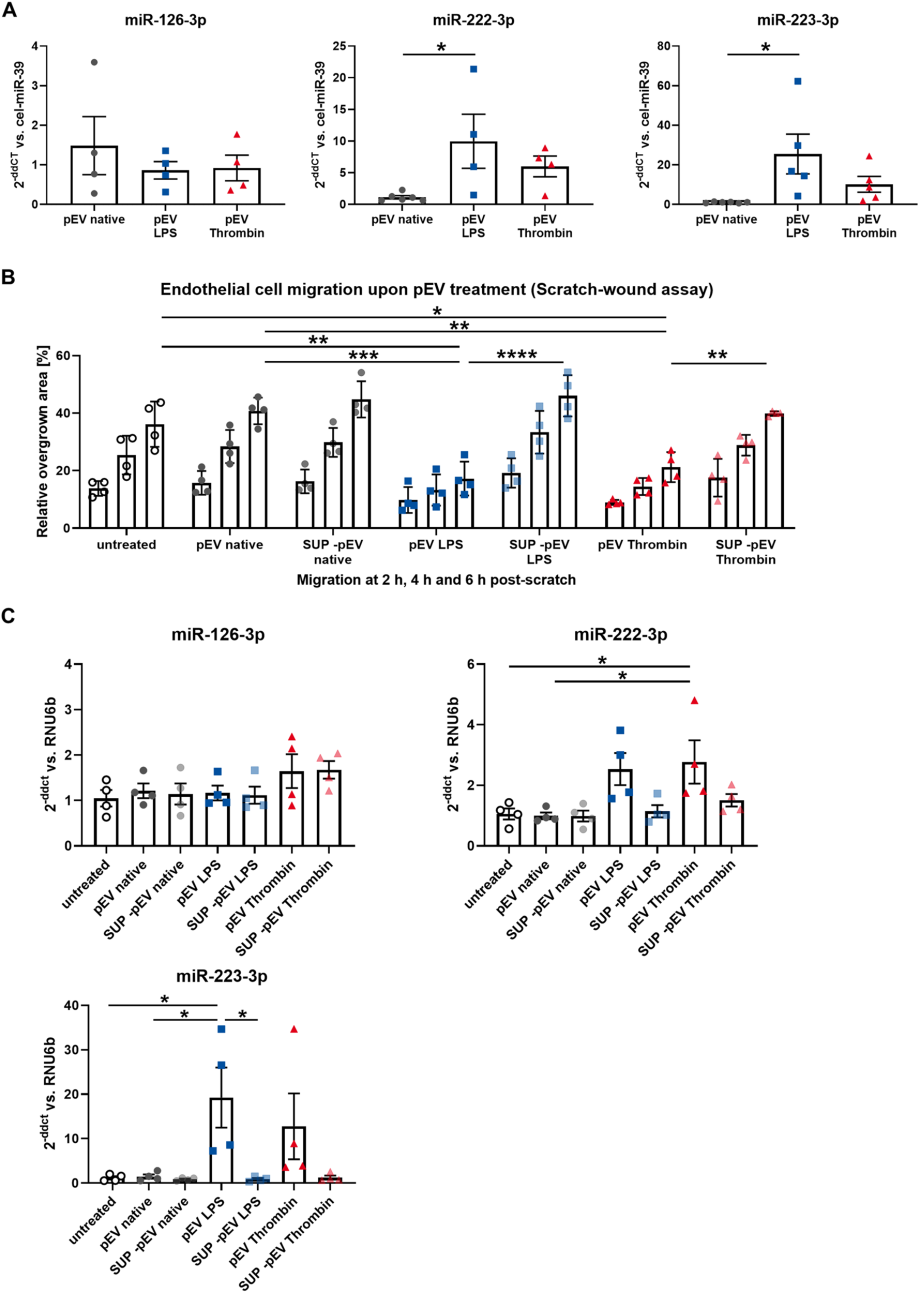
**a** Quantification of miR-126-3p, miR-222-3p, and miR-223-3p in pEVs derived from native platelets and after stimulation with LPS or thrombin by qPCR. miR levels are expressed as 2^−ddCT^ vs. cel-miR-39, which was added as a spike-in reference, *n* = 4–6. **b** Endothelial cell migration was assessed in a scratch–wound assay after stimulation with the respective pEVs or pEV-depleted supernatants, *n* = 4. **c** Quantification of miR-126-3p, miR-222-3p, and miR-223-3p in HCAECs after stimulation with pEVs or pEV-depleted supernatants. miR levels are expressed as 2^−ddCT^ vs RNU6b, *n* = 4. Data are presented as individuals with the mean ± SEM; *p < 0.05, **p < 0.01, ***p < 0.001, ****p < 0.0001, ANOVA + Tukey’s multiple comparison test

As a next step, we wanted to further characterize the biological function of in vitro-generated, large platelet-EVs (pEVs) upon activation. To this end, platelets were isolated from healthy donors and used to generate pEVs in vitro, with and without prior stimulation with LPS or thrombin. In the pEVs, we measured the levels of three functionally important and highly abundant microRNAs by qPCR: miR-126-3p [41], miR-222-3p [42], miR-223-3p [40, 43]. We found that vesicular miR-222-3p and miR-223-3p levels were significantly increased after stimulation with LPS, while activation with thrombin only lead to less-pronounced and, therefore, non-significant changes. The levels of miR-126-3p in the pEVs remained unchanged across the conditions (Fig. 5A). To test the impact of LPS and thrombin activation on pEV function, we incubated the pEVs with otherwise native HCAECs and performed a migration assay. Endothelial regeneration is an important factor involved in left atrial thrombus formation and thus particularly relevant in this context [9]. We found that both, LPS and thrombin activation of platelets, induced the release of pEVs, which significantly inhibited endothelial cell migration. No differences were detected upon incubation with native pEVs or with supernatants depleted of pEVs. These findings were mirrored by increased levels of miR-222-3p and miR-223 in the recipient HCAECs after treatment with pEVs from thrombin- and LPS-activated platelets, respectively. Endothelial miR-126-3p levels remained unchanged by pEV treatment.

**Fig. 6:**
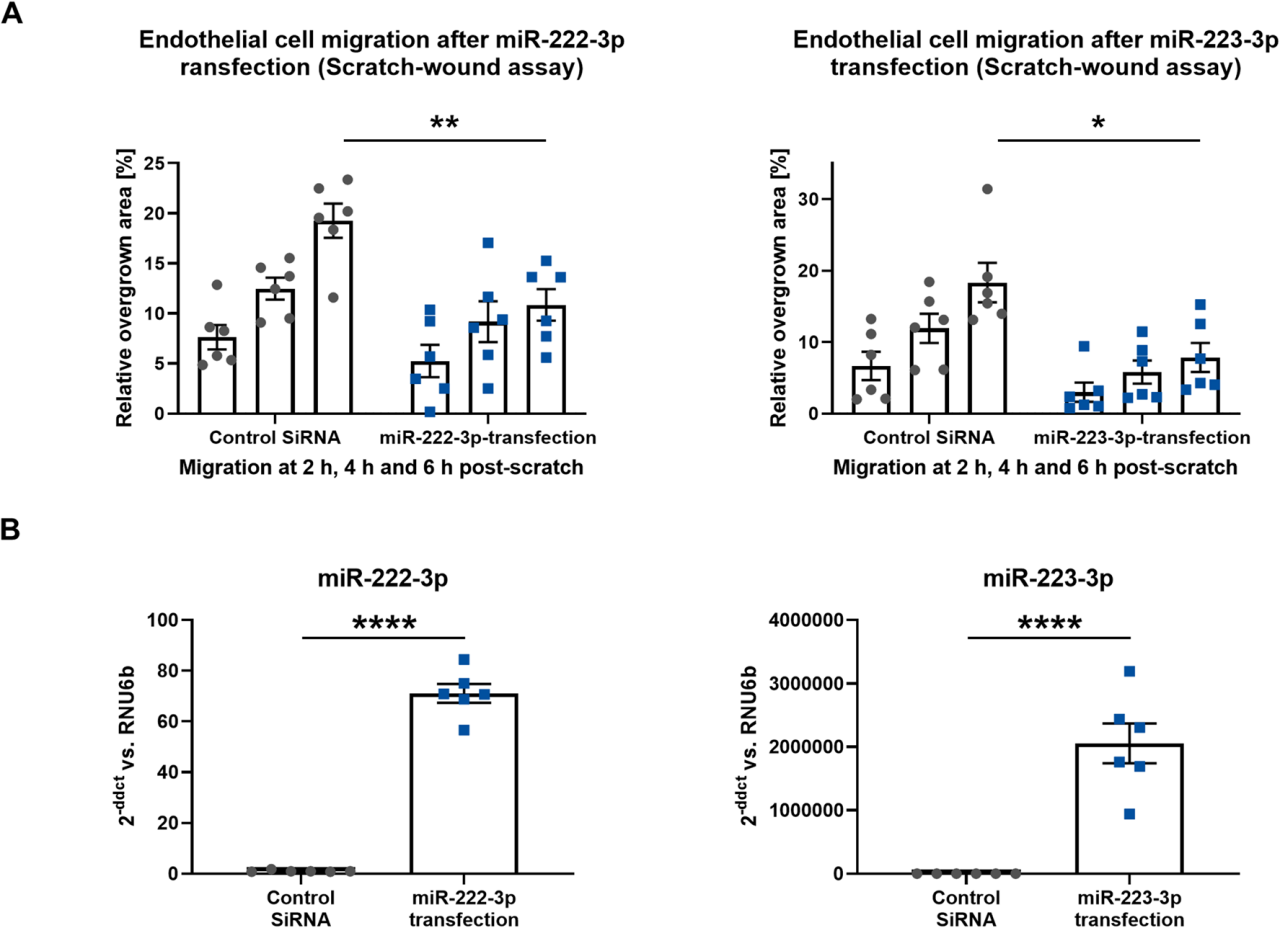
**a** Endothelial cell migration was assessed in a scratch–wound assay after transfection of miR-222-3p and miR-223-3p, *n* = 6. **b** Quantification of miR-222-3p, and miR-223-3p in HCAECs after transfection of miR-222-3p and miR-223-3p. miR levels are expressed as 2^−ddCT^ vs RNU6b, *n* = 6. Data are presented as individuals with the mean ± SEM; *p < 0.05, **p < 0.01, ****p < 0.0001, unpaired *t* test

The anti-angiogenic potential of miR-222-3p and miR-223-3p was confirmed in a transfection experiment. We found HCAEC migration to be significantly reduced after experimental overexpression of miR-222-3p as well as miR-223-3p (Fig. 6A, B).

## Discussion

In this study, we show for the first time, that the type of atrial fibrillation present in a patient has an effect on the distribution of lEV subtypes in the left atrial appendage. In vitro*,* we found that EVs from activated platelets exhibit significantly higher levels of miR-222-3p and miR-223-3p and reduce the migratory potential of the endothelial EV-recipient cells.

Previous studies have shown, that the level of platelet-derived lEVs is significantly higher in the peripheral venous blood of non-valvular AF patients, compared to patients who have no history of AF [21]. Similar results were obtained by Azzam and co-workers, who found increased levels of platelet-derived EVs in patients with valvular AF [22]. Ederhy and co-workers found that, besides higher levels of platelet-derived EVs, also endothelial-cell-derived EVs were higher in patients with AF compared to healthy patients. These differences collapsed, however, when AF patients were compared to a patient collective with a similar cardiovascular risk profile but without AF [22]. In our study, we did not analyze peripheral venous blood, but instead we found elevated levels of platelet-derived EVs in blood from the RA of AF patients, which is the closest location to peripheral veins that we tested. In our study, endothelial-cell-derived EVs were lower in the RA of AF patients. When comparing the baseline characteristics of the groups in our study, no significant differences were detected for most cardiovascular risk factors, age, or other confounding factors, such as kidney disease, anemia, heart failure, coronary artery disease, and valvular defects (Tables 1, 2). Only the prevalence of diabetes was higher in the control group, which is unlikely to explain why EV levels in the AF group were higher [44].

A very recent study also analyzed EV in the left atrial appendage in AF patients [24]. However, different from our analysis, the blood for extracting the EVs was taken during open heart surgery, a procedure that is very invasive and may itself cause activation of various blood cells. Furthermore, the authors used an EV array to distinguish between the EV subtypes, which does not allow for the analysis of EVs on a single particle basis. Interestingly, it was found that tissue factor bearing EVs, were significantly increased in the LAA in AF patients. Tissue factor bearing EVs had previously shown to be involved in thrombosis and to be particularly relevant in the context of malignant diseases [45–47].

In another very interesting study, Pourtau and co-workers found a decrease in the pro-coagulatory activity of EVs in the LA after induction of AF [23]. These results were attributed to increased consumption of the EVs during AF by coagulation, however, no measurements of EV levels were performed in the left atrium.

From the data in our study, we can exclude that the higher proportion of platelet-derived EVs in the LAA are caused by higher levels of platelets in the AF patients, because no differences in platelet levels were detected between the groups. Therefore, it seems plausible, that the differences are the result of increased platelet activation and EV release in the LAA of AF patients. This would be in line with previous reports of platelet activation leading to the release of EVs [40].

The effect of platelet activation on platelet-derived EV function was further investigated in vitro*.* Endothelial-cell function and endothelial healing have been implicated in atrial thrombus formation [9, 10]. Therefore, we were interested in three miRs, which are known to strongly influence endothelial-cell function: miR-126-3p is known to promote endothelial regeneration [48], and miR-222-3p and miR-223-3p inhibit endothelial regeneration [49, 50]. After platelet activation, the released EVs contained significantly higher levels of the anti-regenerative miR-222-3p and miR-223-3p. The EVs were loaded with anti-angiogenic miR-222-3p and miR-223-3p, which then caused the regenerative capacity to be lower in endothelial EV-recipient cells. This endothelial phenotype after EV stimulation was accompanied by increased mir-222-3p and miR-222-3p levels in the endothelial EV-recipient cells. These results confirm the previous findings that platelet activation can increase vesicular miR-223-3p transfer to endothelial recipient cells in a functionally relevant manner [51].

Unfortunately, our study also has some limitations. The number of the patients we included in our study is higher than in most other investigations of lEV levels in AF patients, but still some differences in the EV levels of less abundant EVs, such as endothelial-cell-derived EVs, might not have been detected due to insufficient power.

In summary, we provide for the first evidence that the relative amount of platelet-derived EVs is higher in the left atrial appendage in patients with permanent AF, compared to patients with non-permanent AF. This may lead to increased transfer of miR-223-3p into endothelial cells and consequently to reduced endothelial healing, which has been shown to be thrombogenic itself. This work can therefore serve as a basis for further investigations of the role of EVs in thrombus formation, specifically in the milieu of the left atrial appendage.

## Supplementary Information

Below is the link to the electronic supplementary material.

Supplementary file1 Fig. S1 Dilution curves of different lEV subtypes in flow cytometry. (DOCX 453 kb)

## Data Availability

Underlying data will be made available by the corresponding authors upon reasonable request.
