# Supplementary figures and images for: Large extracellular vesicles in the left atrial appendage in patients with atrial fibrillation—the missing link?
Source: Clin Res Cardiol. 2021 Jun 1;111(1):34–49. doi: 10.1007/s00392-021-01873-4 (PMC8766378; doi:10.1007/s00392-021-01873-4)

###
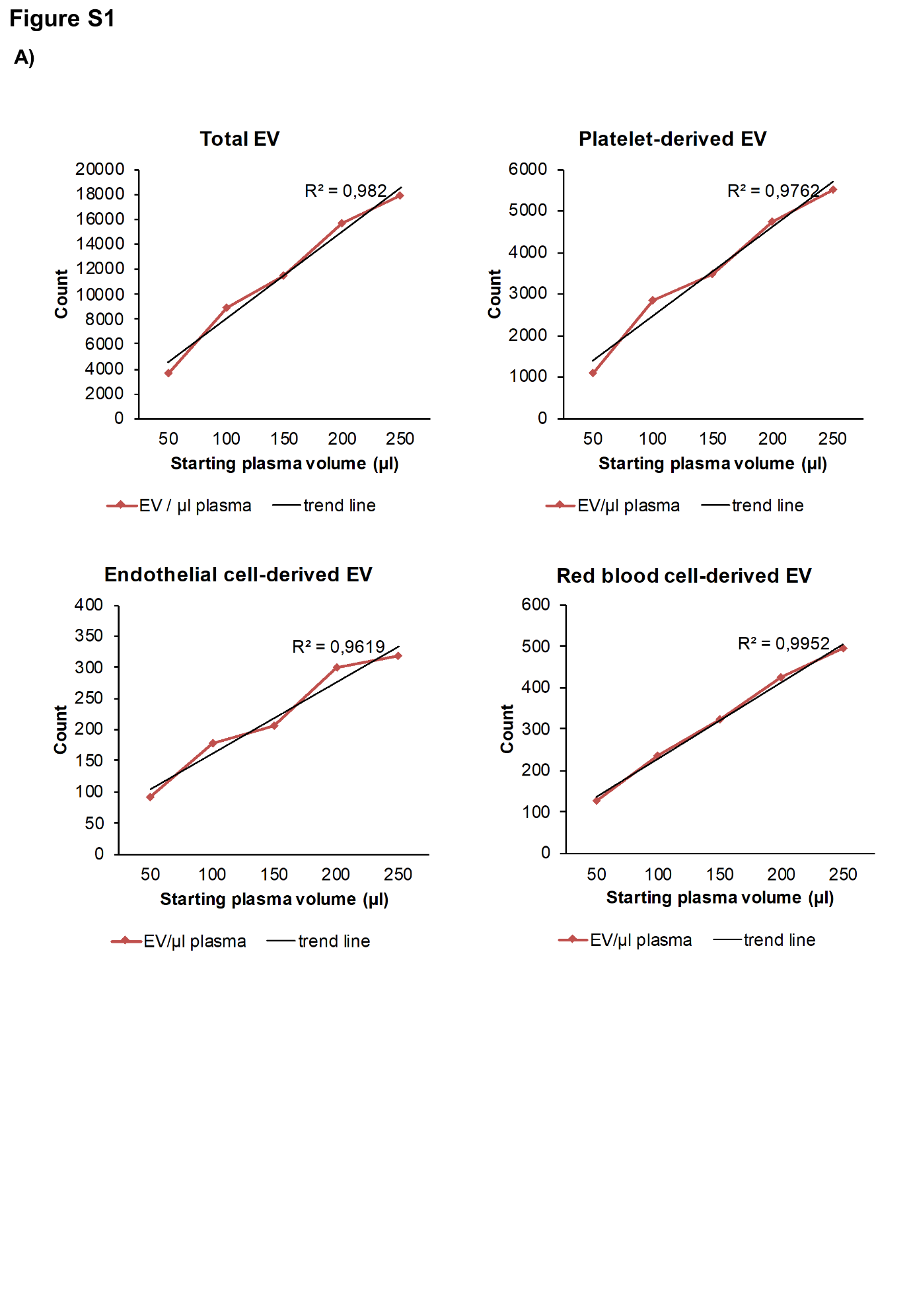


### **Figure S1:**

Dilution curves of different lEV subtypes in flow cytometry

Supplement: Supplementary file 1 — Supplementary file1 Fig. S1 Dilution curves of different lEV subtypes in flow cytometry. (DOCX 453 kb) [file 392_2021_1873_MOESM1_ESM.docx]
